# VirB8-like protein TraH is crucial for DNA transfer in *Enterococcus faecalis*

**DOI:** 10.1038/srep24643

**Published:** 2016-04-22

**Authors:** Christian Fercher, Ines Probst, Verena Kohler, Nikolaus Goessweiner-Mohr, Karsten Arends, Elisabeth Grohmann, Klaus Zangger, N. Helge Meyer, Walter Keller

**Affiliations:** 1 Institute of Molecular Biosciences, NAWI Graz, University of Graz, Austria; 2 Division of Infectious Diseases, University Medical Center Freiburg, Germany; 3 Faculty of Biology, Microbiology, Albert-Ludwigs-University Freiburg, Germany; 4 Center for Structural System Biology (CSSB), University Medical Center Hamburg-Eppendorf (UKE), Hamburg, Germany; 5 Deutsches Elektronen-Synchrotron (DESY), Hamburg, Germany; 6 Institute of Molecular Biotechnology (IMBA), Austrian Academy of Sciences, Vienna, Austria; 7 Research Institute of Molecular Pathology (IMP), Vienna, Austria; 8 Robert Koch Institute, Berlin, Germany; 9 Beuth University of Applied Sciences, Berlin, Germany; 10 Institute of Chemistry, University of Graz, Graz, Austria; 11 Department of General and Visceral Surgery, University of Oldenburg, Germany

## Abstract

Untreatable bacterial infections caused by a perpetual increase of antibiotic resistant strains represent a serious threat to human healthcare in the 21^st^ century. Conjugative DNA transfer is the most important mechanism for antibiotic resistance and virulence gene dissemination among bacteria and is mediated by a protein complex, known as type IV secretion system (T4SS). The core of the T4SS is a multiprotein complex that spans the bacterial envelope as a channel for macromolecular secretion. We report the NMR structure and functional characterization of the transfer protein TraH encoded by the conjugative Gram-positive broad-host range plasmid pIP501. The structure exhibits a striking similarity to VirB8 proteins of Gram-negative secretion systems where they play an essential role in the scaffold of the secretion machinery. Considering TraM as the first VirB8-like protein discovered in pIP501, TraH represents the second protein affiliated with this family in the respective transfer operon. A markerless *traH* deletion in pIP501 resulted in a total loss of transfer in *Enterococcus faecalis* as compared with the pIP501 wild type (wt) plasmid, demonstrating that TraH is essential for pIP501 mediated conjugation. Moreover, oligomerization state and topology of TraH in the native membrane were determined providing insights in molecular organization of a Gram-positive T4SS.

Prokaryotic genome plasticity and therefore evolutionary success greatly relies on a variety of independent mechanisms congruously summarized under the term of horizontal gene transfer (HGT)^1^,^2^. While transduction (mediated by bacteriophages) and transformation (unspecific uptake of extracellular DNA) are usually associated with random modifications, the event of bacterial conjugation represents a directed and specific mechanism for genetic dissemination^3^,^4^,^5^,^6^,^7^. In fact, conjugation is the primary pathway for the spreading of antibiotic resistance and virulence genes among microbial communities^2^. The underlying molecular machinery, also known as T4SS, mediates a variety of functions such as the uptake and release of DNA and the translocation of effector proteins^8^,^9^. T4SSs play an essential role in virulence of several human pathogens, such as *Bordetella pertussis, Campylobacter jejuni, Legionella pneumophila, Salmonella typhi, Helicobacter pylori*, as well as commensal and pathogenic *Escherichia coli* and the plant pathogen *Agrobacterium tumefaciens*^10^,^11^,^12^. Despite the clinical relevance of numerous G+ pathogens (e.g. enterococci, staphylococci, streptococci, bacilli, clostridiae), most information available for this group originates from comparative studies on Gram-negative (G−) model organisms^13^,^14^,^15^,^16^,^17^,^18^. Driven by an increasing number of fatal bacterial infections in nosocomial environments associated with the growing inefficiency of antibiotic drugs towards multi-resistant species, G+ organisms start to attract increasing attention^19^,^20^,^21^. Structural and functional studies on distinct transfer proteins from the conjugative plasmid pIP501 have contributed to a better understanding of pIP501 mediated conjugation. The multiple antibiotic-resistance plasmid is frequently isolated from clinical *E. faecalis* and *E. faecium* strains and serves as a G+ model system mainly due to its small size and surprisingly broad-host range^22^. The pIP501 transfer system is encoded within a single operon and consists of 15 distinct transfer proteins (TraA-TraO)^23^,^24^. The muramidase TraG was functionally characterized and found to be essential for the DNA transfer by locally degrading the peptidoglycan layer of the cell^25^. The extracellular C-terminal domain of the bitopic transmembrane (TM) protein TraM was identified as VirB8-like, while the 3.0 Å structure of TraK exhibits a novel fold^26^,^27^. Very recently, the structure of the double stranded DNA binding protein TraN became available. The fold resembled transpositional excisionases as well as transcriptional regulators of the MerR family. Employing a novel sequencing-based DNA footprinting assay, the respective TraN binding site upstream of the pIP501 *nic-*site was mapped, suggesting a possible role as auxiliary factor for the relaxase TraA^28^. Currently available knowledge about G+ T4SSs has been summarized by Goessweiner-Mohr *et al*.^16^. Structural and functional knowledge of TraH, however, remained elusive.

In this study, we present the NMR solution structure of the soluble domain of the essential 21.2 kDa transfer protein TraH (formerly ORF8; GenBank CAD44388.1) along with a detailed biophysical and biochemical characterization of its putative role in the conjugative conduit. Localization studies revealed that the bitopic TM protein localizes to the cell wall via a hydrophobic N-terminal domain. *In vivo* protease protection assays on *E. faecalis* protoplasts confirmed the predicted topology where the functional C-terminal domain faces the cytoplasm. Comparative 3D-structure alignments of the domain have resulted in the functional assignment of TraH to the VirB8 family (Pfam: PF04335). Although N-terminally truncated TraH always appears as monomer in solution, size exclusion chromatography, *in vitro* and *in vivo* crosslinking studies and semi-native PAGE analysis suggested oligomerization of the full-length protein. Deletion of *traH* in pIP501 leads to a complete loss of transfer efficiency, implying that TraH is an essential player in G+ bacterial conjugation.

## Results

### Biophysical and structural characterization of TraH

**Figure 1:**
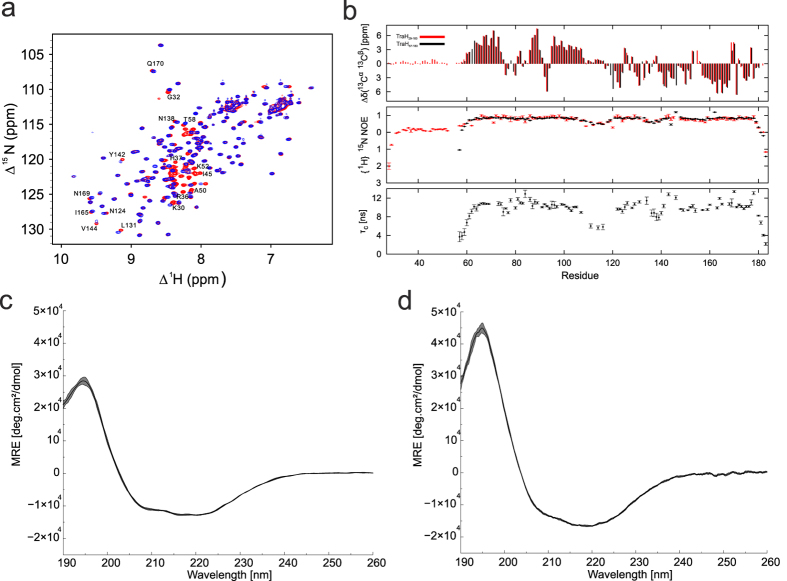
Structural integrity of TraH variants. (**a**) HSQC spectra overlay of TraH_29–183_ (in red) and TraH_57–183_ (in blue). (**b**) ^13^C secondary chemical shifts (upper panel), {^1^H}-^15^N heteronuclear chemical shifts (middle panel) and apparent rotational correlation time τ_c_ for TraH_57–183_ (in black) and TraH_29–183_ (in red) are plotted versus the residue number. CD-spectrum of TraH_29–183_ (**c**) and TraH_57–183_ (**d**) between 190 and 260 nm. The mean of 10 individual wavelength scans is represented by a black line. The standard deviation is displayed by a grey shaded area.

Initial *in silico* analysis of TraH amino acid sequence (21.2 kDa) predicted an N-terminal hydrophobic region spanning from Val16 to Gln31^29^. Deletion of the N-terminus facilitated the expression and purification of the protein in *E. coli*. We worked with two truncation variants, TraH_29–183_ (18.4 kDa) and TraH_57–183_ (14.9 kDa). Both proteins are soluble and well folded up to a concentration of 700 μM, as indicated by ^15^N^1^H-HSQC spectra (Fig. 1a).

NMR secondary chemical shift analysis indicated that the N-terminal residues 28–58 adopt a random coil conformation, followed by three α-helices, which are connected by short loops. The C-terminal part of the protein forms a β-sheet (Fig. 1b upper panel). This is consistent with CD analysis, which predicts a mixed α/β secondary structure composition with a high content of unstructured regions in TraH_29–183_ (Fig. 1c). However, the content of random coil conformation is reduced in TraH_57–183_ (Fig. 1d) corroborating that TraH_57–183_ constitutes the folded core of the protein. Indeed, {^1^H}-^15^N NOEs revealed that the N-terminal residues 28–58 are highly flexible, as indicated by heteronuclear NOE values, which are negative or close to zero. In addition, C-terminal residues 180–183 show increased conformational flexibility (Fig. 1b – middle and lower panel).

**Figure 2:**
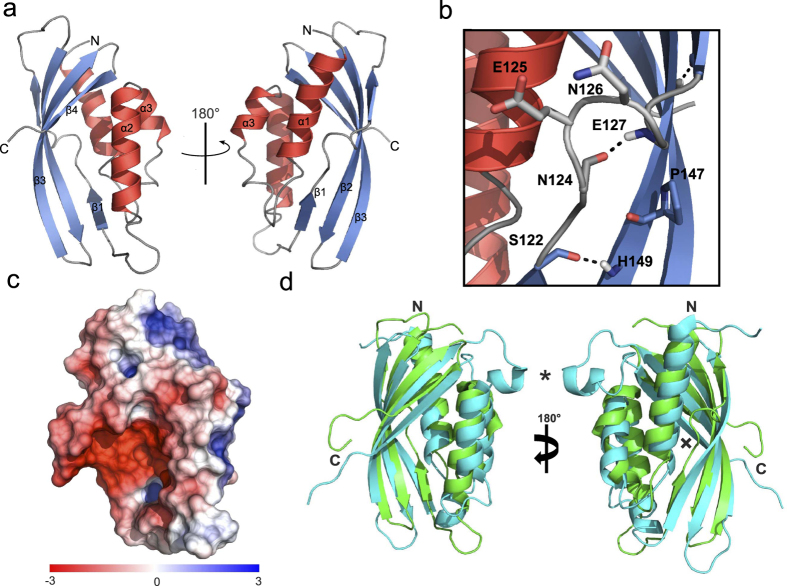
NMR solution structure of TraH_57–183_. (**a**) The soluble domain of TraH consists of three N-terminal α-helices (red) and a highly curved C-terminal β-sheet (blue) made up by essentially 4 β-strands. (**b**) The first strand (β1) is interrupted by the insertion of two amino acids at position 125–126. As a consequence, amino acids E125 and N126 are looped out of the β-strand due to steric hindrance splitting the first into β1 and β1′. The hydrogen bond network between β1/β1′ and β2 is further perturbed by a proline at position 147 in β2. (**c**) Surface representation of TraH_57–183_ indicating a polarized positive (blue) and negative (red) surface charge distribution. (**d**) Representative structural alignment of TraH_57–183_ in green and VirB8 from *A. tumefaciens* (PDB: 2CC3) in cyan. Obvious discrepancies between TraH and classical VirB8-like proteins are marked with an “*” (helical loop insertion) and an “**x**” (interrupted β1–β1′ strand), respectively.

To functionally characterize the protein we determined the solution structure of TraH_57–183_. The protein consists of three N-terminal antiparallel α-helices (α1, α2 and α3) and a highly curved β-sheet comprising four antiparallel β-strands (β1, β1′, β2 and β3) at the C-terminus (Fig. 2a; see also Supplementary Video S1). The first strand is interrupted due to the presence of two amino acids at position 125–126. As a consequence, residues Glu125 and Asn126 are looped out of the β-strand due to steric hindrance, splitting the first strand into two sub-strands (β1, β1′). Furthermore, the hydrogen bond network between β1/β1′ and β2 is interrupted as Pro147 in β2 does not provide a hydrogen bond donor (Fig. 2b). Looping out of amino acids Glu125-Asn126 is evident both from backbone dihedral angles predicted from chemical shifts with TALOS+ and from the NOE pattern, which do not show the characteristics typical for β-strands (Supplementary Fig. S1). Additionally, a classic antiparallel β-bulge is formed by residues Gln166 (residue X), Thr173 (residue 1) and Gln174 (residue 2)^30^. The β-sheet wraps around one face of helix α1. Helices α2 and α3 are tightly packed against each other thus shaping one edge of a deep, negatively charged surface groove with β3 and β4, forming the other edge (Fig. 2c). Refinement statistics for the TraH solution structure can be found in Supplementary Table S1.

### TraH is a member of the VirB8 family

A structure-based homology search revealed that TraH is related to proteins of the VirB8 family, which all exhibit a NTF2-like fold. Despite very low sequence identity (6–19%), the structures of VirB8-like T4SS proteins of G- *Brucella suis (*PDB code 2BHM, 10% sequence identity), *Bartonella grahamii* (PDB code 4NHF, 14% sequence identity), *Bartonella quintana* (PDB code 4LSO, 6% sequence identity), *Rickettsia typhi* (PDB code 4O3V, 19% sequence identity) and *A. tumefaciens* (PDB code 2CC3, 10% sequence identity) are highly similar with backbone RMSD values ranging between 2.6–2.8 Å (Supplementary Table S2). The most prominent differences in the individual structures comprise of an extended loop and a short α-helix inserted between β3 and β4 of the curved β-sheet in VirB8, which is absent in TraH (Fig. 2d – indicated by^*^). Moreover, almost all analyzed VirB8 proteins but not TraH contain a complete first β-strand (β1) with a consecutive hydrogen-bond network to β2 (Fig. 2d – indicated by X). Nevertheless, the overall curvature of the β-sheet in TraH is not affected by the interruption of the β1/β1′ strand.

Surprisingly, the TraH structure more closely resembles G- VirB8 members than the C-terminal domains of the G+ proteins TraM_pIP501_ (PDB code 4EC6) and TcpC_pCW3_ (PDB code 3UB1). As prominent members of their respective subclasses, both proteins were found to form trimers in the crystal and possess an N-terminal coiled-coil motif. According to a new classification system of VirB8-like proteins proposed by Goessweiner-Mohr *et al*.^16^, TraH can be affiliated with the ALPHA subclass while TcpC_pCW3_ represents the prototype for the BETA subclass and TraM_214–322_ belongs to the GAMMA subclass^27^.

### TraH is N-terminally anchored to the *E. faecalis* cell envelope

**Figure 3:**
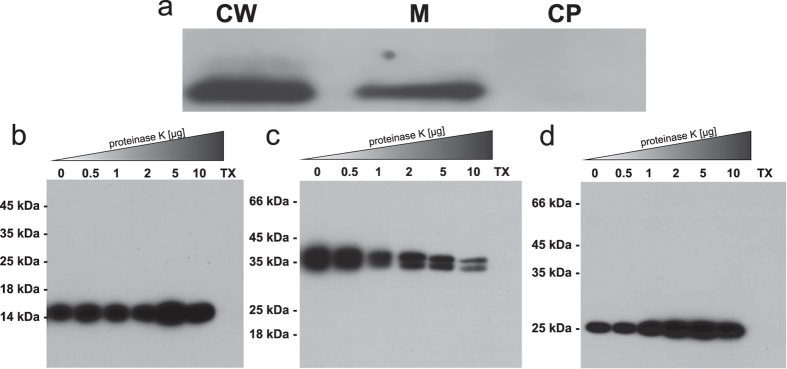
Cellular localization and topology of TraH. (**a**) TraH localizes to the cell envelope of *E. faecalis* JH2-2 cells harboring pIP501. CW_ cell wall; M_ membrane; CP_ cytoplasm. (**b**–**d**) Protease protection assay with *E. faecalis* JH2-2 (pIP501) protoplasts exposed to increasing amounts of proteinase K. The cytoplasmic transfer protein TraN (**b**) and the membrane anchored extracellular protein TraK (**c**) analyzed in the same sample serve as intra- and extracellular controls, respectively. TraH (**d**) and TraN resist proteolytic degradation upon protease treatment as both are protected by the lipid bilayer of the cytoplasmic membrane. Contrarily, TraK is gradually degraded. Solubilization of the bacterial membrane with Triton X-100 (TX) leads to a complete depletion of all proteins. The TraK double band arises from utilization of a second start codon and ribosomal binding site within the coding region of the gene which leads to two gene products *in vivo*^26^.

In order to complete our picture of the overall architecture of full length TraH *in vivo*, we analyzed its topology and localization. To determine TraH localization, an exponentially growing culture of *E. faecalis* JH2-2 harboring pIP501 was harvested, washed and fractionated. We exclusively detected the protein in the cell wall and membrane fractions (Fig. 3a).

TraH topology under native conditions was further evaluated using a protease protection assay. In this procedure, extracellular proteins and protein domains are proteolytically digested by the addition of increasing amounts of proteinase K to *E. faecalis* JH2-2 (pIP501) protoplasts. The cytoplasmic protein TraN served as a control for intracellular proteins (Fig. 3b). The extracellularly anchored transfer protein TraK (Fig. 3c) is degraded in a concentration dependent manner, whereas TraN and TraH (Fig. 3d) are stable under the same conditions. Only upon solubilization of the protoplasts with Triton X-100 complete degradation of all three proteins (Fig. 3b–d, lanes TX) was observed. Therefore, we conclude that the C-terminal domain of TraH faces the cytoplasm and is protected from proteolytic degradation by the intact cytoplasmic membrane.

### TraH oligomerization is facilitated by the TM motif

VirB8-like proteins have been shown to function as oligomers in solution^3^,^27^,^31^,^32^,^33^,^34^,^35^. However, an overall apparent rotational correlation time of ~10 ns has been derived from ^15^N R_1_ and R_2_ relaxation rates for TraH_57–183_ (Fig. 1b-lower panel), which is consistent with a monomer. To validate that both TraH truncation variants are monomeric in solution we performed analytical size exclusion chromatography (SEC) and small angle X-ray scattering (SAXS) experiments.

**Figure 4:**
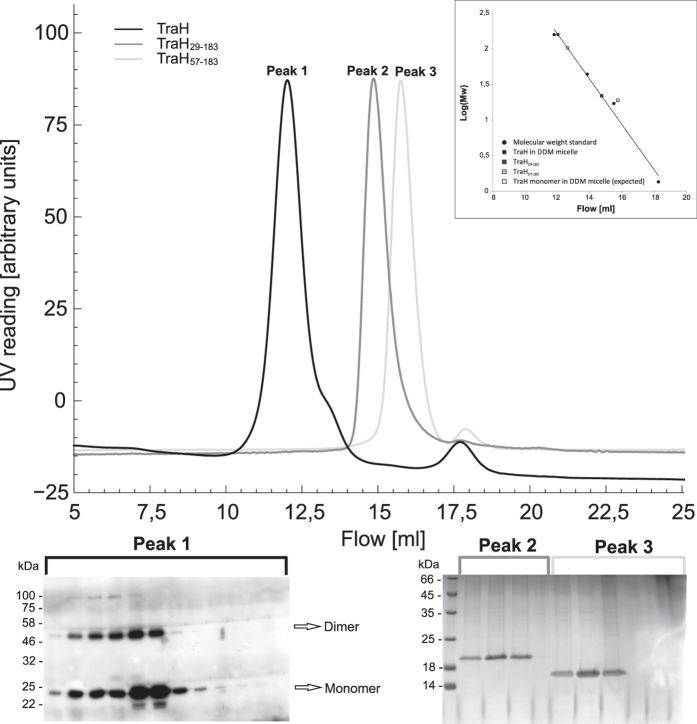
Size exclusion chromatogram of TraH variants. Full length TraH (black), TraH_29–183_ (dark grey) and TraH_57–183_ (light grey) elute as single peaks with an apparent molecular weight of 158, 26 and 19 kDa, respectively. Comparing these values to a standard run with proteins of known size on the same column, both truncations can be considered monomeric in solution (see inlet). Taking an average molecular weight of 70 kDa for a DDM micelle into account, reconstituted full length TraH elutes with an apparent molecular weight of a tetramer. Immuno-blot analysis (TraH, left panel) and Coomassie-stained SDS-PAGE analysis (TraH_29–183_ and TraH_57–183_, right panel) of the individual peak fractions are shown below. Peak heights have been normalized to arbitrary units.

**Figure 5:**
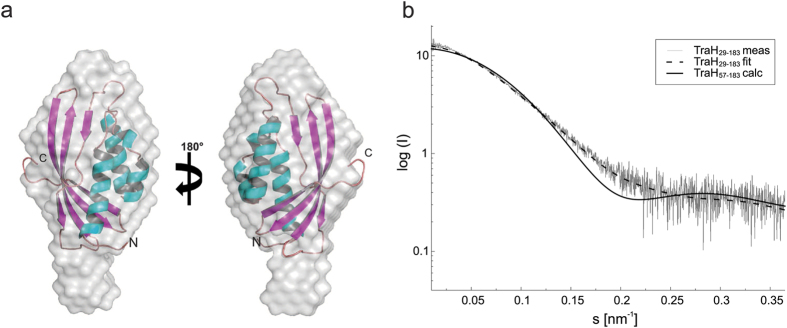
Small angle X-ray scattering experiments of TraH_29–183_. (**a**) Superposition of the NMR solution structure of TraH_57–183_ to the SAXS envelope shape model of TraH_29–183_. (**b**) Experimental scattering curve of the larger construct (dashed line) compared to the calculated scattering curve of TraH_57–183_ (solid line).

In analytical SEC experiments, TraH_29–183_ and TraH_57–183_ eluted as a single peak (Fig. 4) indicative of a homogenous, monomeric species with apparent molecular weights of 26 kDa for TraH_29–183_ and 19 kDa for TraH_57–183_. The molecular weight of TraH_29–183_, derived by SAXS was determined to be 15.1 kDa from guinier analysis and real space calculations, which is clearly consistent with a monomeric form (I_0_ = 12.7; R_g_ = 2.1 nm). Even at high concentration up to 700 μM no significant protein aggregation was observed. 19 calculated discrete *ab initio* models resulted in a particle comprising an additional tail structure projecting from one side of the molecule (Fig. 5a).

For comparison, the theoretical small angle scattering profiles of the TraH_57–183_ NMR structure ensemble were calculated and superimposed on the experimental curve obtained for TraH_29–183_ using the program CRYSOL^36^ (Fig. 5b). Minimizing the normalized spatial discrepancy in an undirected fitting process of the NMR ensemble into the SAXS envelope of TraH_29–183_ results in a superposition of the folded domain with the main density of the truncated N-terminus pointing towards the empty tail of the SAXS model. We conclude that the unstructured N-terminal region of TraH_29–183_ accounts for the elongated shape of the SAXS model also causing the higher apparent molecular weight in SEC experiments.

**Figure 6:**
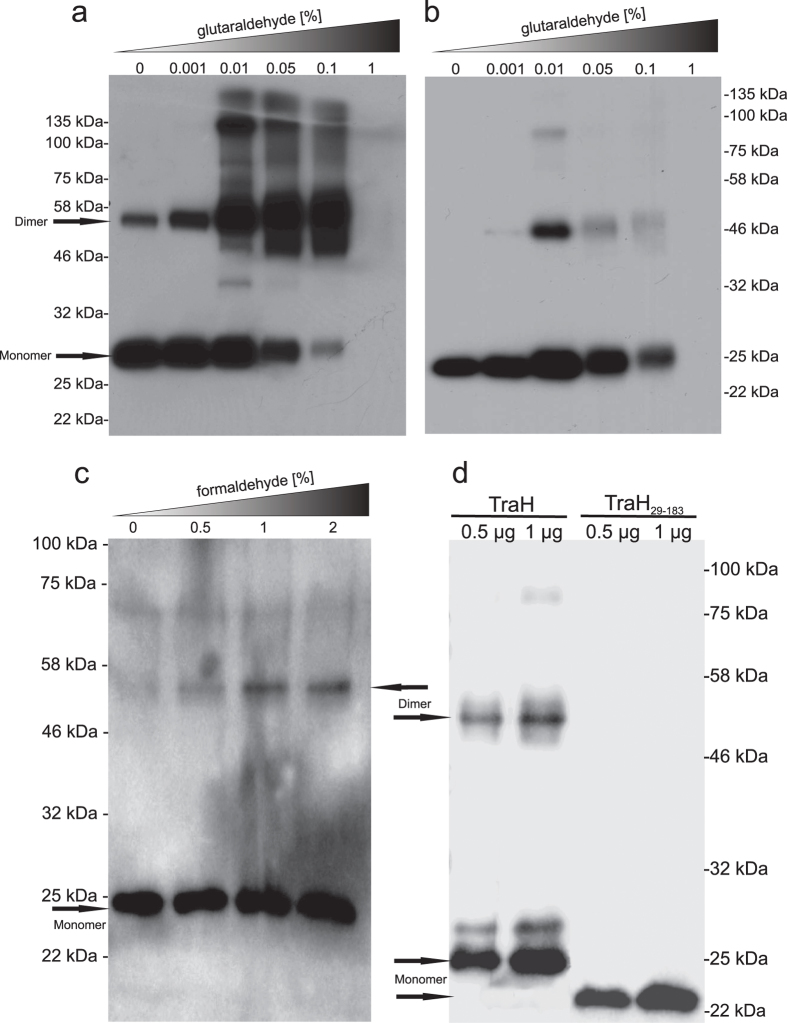
Analysis of the oligomerization state of TraH and TraH_29–183_ by chemical crosslinking and semi-native PAGE experiments. (**a**) Upon addition of increasing GA amounts, monomeric full-length TraH is shifted towards larger aggregates with defined molecular weight. According to a molecular weight standard (22–135 kDa), TraH presumably exists in an equilibrium of monomers, dimers and even tetramers in solution. Interestingly, a small fraction of dimeric TraH was also detected in absence of GA underlining the strength of the interaction. (**b**) Lacking the hydrophobic transmembrane region, TraH_29–183_ shows a significantly reduced ability for forming oligomers in solution. Here, a significant band corresponding to the size of a dimer is only detectable at a GA concentration of 0.01%. Higher amounts of the crosslinking agent led to the formation of unspecific aggregates that did not enter the gel. (**c**) *In vivo* FA crosslinking of *E. faecalis* cells constitutively expressing TraH. (**d**) Semi-native PAGE analysis of purified TraH and TraH_29–183_.

In addition, oligomerization of full length TraH was investigated *in vitro* using glutaraldehyde (GA) crosslinking of the purified protein as well as *in vivo* by formaldehyde (FA) crosslinking of intact *E. faecalis* cells constitutively expressing TraH. According to western blot analysis, TraH forms SDS stable dimers even in the absence of GA. Increasing the GA concentration successively led to a shift of the monomeric species towards defined dimeric and even higher molecular weight aggregates (Fig. 6a). In contrast, a significant dimeric fraction was only detectable at 0.01% GA concentration employing TraH_29–183_ in the same experiment (Fig. 6b). 1% GA led to the formation of unspecific aggregates incapable of entering the SDS-PAGE gel. Similarly, TraH was also found to exist as dimer in the membrane environment as shown by *in vivo* crosslinking experiments applying increasing amounts of FA on *E. faecalis* (pEU327-RBS-*traH*) cells (Fig. 6c). This is largely consistent with analytical SEC of full length TraH reconstituted in *n*-dodecyl-β-D-maltopyranoside (DDM) micelles. Taking an average micellar molecular weight of about 70 kDa^37^,^38^ into account, the majority of TraH elutes with a retention volume corresponding to an apparent molecular weight of about 80–90 kDa, corresponding to a tetramer. To further investigate the oligomeric state of the protein and resolve discrepancies between SEC and GA experiments, we performed a semi-native PAGE with purified TraH and TraH_29–183_. As evident from western blot analysis, a band corresponding to the size of a TraH dimer is only detectable for the full-length protein but not for the truncation derivative employing the purified proteins in an *in vitro* set-up (Fig. 6d). Consequently, the apparently higher molecular weight derived from SEC experiments might either be due to the presence of two TraH dimers within one DDM micelle or, more likely, due to the increased hydrodynamic radius of the elongated protein-detergent complex. Thus, we conclude that the TM motif present in full-length TraH is essential for the formation of defined oligomers in solution.

### TraH is an essential component in pIP501-mediated conjugation

The impact of the *traH* deletion on pIP501 transfer was studied by biparental matings. Matings were performed with *E. faecalis* JH2-2 (pIP501), *E. faecalis* JH2-2 (pIP501∆*traH*) and *E. faecalis* JH2-2 (pIP501∆*traH* complemented with pEU327-RBS-*traH*) as donor strains, and *E. faecalis* OG1X as recipient. *E. faecalis* JH2-2 (pIP501) arose from the same merodiploid *E. faecalis* JH2-2 (pIP501::pKA∆*traH*) strain as the *traH* knockout mutant *E. faecalis* JH2-2 (pIP501∆*traH*). Transfer rates of this merodiploid-derived *E. faecalis* JH2-2 (pIP501) were identical to that of the wt strain (2.3 × 10^−5^ ± 2.5 × 10^−7^ transconjugants per recipient).

In comparison to *E. faecalis* JH2-2 (pIP501), transfer rates of *E. faecalis* JH2-2 (pIP501∆*traH*) dropped by three orders of magnitude below the detection limit of the assay (<2.3 × 10^−8^ ± 7.9 × 10^−10^ transconjugants per recipient) pointing to an essential role of *traH* in the T4S process.

To complement the *traH* knockout, we generated *E. faecalis* JH2-2 (pIP501∆*traH*, pEU327-RBS-*traH*) with the *traH* wt gene *in trans* on expression vector pEU327. Biparental matings with *E. faecalis* JH2-2 (pIP501∆*traH*, pEU327-RBS-*traH*) as donor and *E. faecalis* OG1X as recipient showed full recovery of transfer capacity, as rates virtually identical to pIP501 wt transfer rates were observed (Supplementary Table S3). Thus, polar effects of the *traH* deletion on downstream *tra* genes could be excluded. The empty pEU327 vector (without *tra* gene) had no effect on pIP501 transfer as demonstrated in Arends *et al*.^25^.

## Discussion

T4SSs function as elaborated transport machines dedicated to the trafficking of proteins or DNA-protein complexes across the bacterial envelope into eukaryotic host cells or bacterial recipients. While the conjugative transfer of DNA significantly increases prokaryotic genome plasticity in the course of evolution, effects on human health are usually adverse. This holds especially true for the exchange of virulence or antibiotic resistance genes that directly or indirectly contribute to a protracted disposition of a pathogen in the human host^9^,^16^,^39^.

Properties of individual T4SS key players have been recently reviewed and could be assigned to functional subgroups^40^. These efforts eventually culminated in the structural characterization of an assembled T4SS of the conjugative *E. coli* plasmid R388, summarized in a series of elegant publications^41^,^42^,^43^. While the outer membrane complex (OMC) is composed of the lipoprotein VirB7 and the C-terminal domains of VirB9 and VirB10 in earlier EM and crystallographic studies, the architecture of the inner membrane complex (IMC) remains partly unclear^41^,^43^,^44^. Consistent with former observations that the T4SS-associated ATPase VirB4_pKM101_ is bound to the VirB7/VirB9/VirB10 core complex, immunogold labelling experiments confirmed its presence in the IMC^45^. In contrast, VirB8_pKM101_ co-purified with other complex components but its location was not probed within the inner membrane translocon. ^125^Iodine labelling determined a stoichiometry of 12 VirB8 molecules to be present in the complex where they might play an important role in complex assembly and initial DNA substrate transport^46^. In general, VirB8 homologs have been found to be crucial structural and functional components of their respective systems^47^,^48^,^49^,^50^,^51^,^52^,^53^. Available crystal structures of soluble VirB8 domains originating from G- (e.g. PDB codes: 2CC3, 2BHM, 4KZ1, 4NHF) and G+ (e.g. PDB codes: 3UB1, 4EC6) T4SSs along with mutational analysis suggested that oligomerization is physiologically relevant^3^,^27^,^33^,^34^,^54^,^55^.

In this study, we present a biophysical and biochemical analysis along with the NMR solution structure of TraH, an essential transfer protein encoded by the G+ conjugative, antibiotic resistance plasmid pIP501. TraH is a membrane-associated protein with a short, negligible N-terminal stretch followed by a TM helix and a large C-terminal domain. Surprisingly and despite very low sequence identity with other transfer proteins (Supplementary Table S2), the structure of TraH resembles those of G- VirB8 molecules. This protein class shares a common NTF2-like domain which, in case of G- members, localizes to the periplasm of the respective host^47^. In contrast, TraH resisted proteolytic degradation in protease protection assays using *E. faecalis* protoplasts, suggesting a reversed topology where the C-terminal domain faces the cytoplasm and not the extracellular environment. This technique has generally proven to provide a valuable tool in unambiguously determining the topology and surface accessibility of transfer proteins in their native environment^26^. Previously, the extracellular C-terminal domain of TraM was identified as the only pIP501 transfer protein comprising a VirB8-like fold. Despite structural analogy of the VirB8-like domains of TraH and TraM, TraH seems to be more related to classical VirB8 members found in G- species. According to a new classification system for VirB8 domain(s) consisting proteins, TraH can be affiliated with the ALPHA subclass while TraM belongs to the GAMMA class^27^. Variations are mainly located in the C-terminal half of TraH, where β-strand β1 splits into two distinct strands (β1 and β1′) due to the presence of residues Glu125 and Asn126. Additionally, Pro147 in the consecutive strand β2 does not provide a hydrogen bond donor, which further prevents association of the two strands at this position. However, the overall curvature of the sheet is not affected. So far, only the recently available structure of the VirB8 homolog DotI derived from the type 4B secretion system of *L. pneumophila* exhibits a similar feature^35^. Furthermore, TraH lacks the conserved NPXG motif between β3 and β4, which is responsible for the helical loop insertion found in virtually all analyzed α-VirB8 members. This motif acts as an important site for protein-protein interactions during the formation of homodimers and is stabilized in a rather rigid conformation throughout that process^3^,^34^. Homodimer formation of TraH rather relies on the presence of the TM helix whereas the absence of the helical loop insertion does not seem to interfere with oligomerization according to protein crosslinking studies. This observation supports the idea that residues in and around the TM motif might play an integral role during self-association of α-VirB8-like proteins as shown for VirB8 from *B. suis* and its homolog TraJ from pSB102^56^. In 2011, it was further demonstrated that exchanging a single amino acid in this domain may have negative effects on homodimer stability and virulence. A role in modulating the strength of VirB8 self-interaction was therefore anticipated^54^.

**Figure 7:**
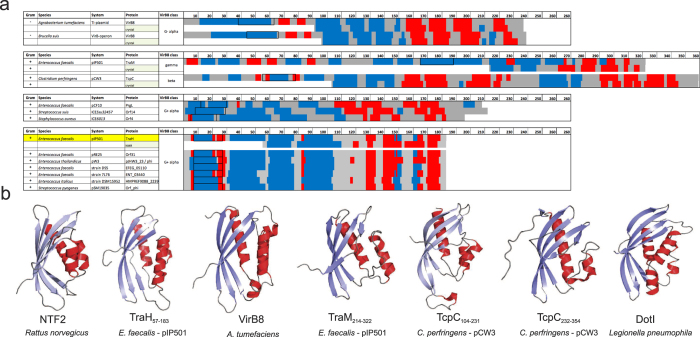
Secondary structure-based comparison of VirB8-like proteins and structural comparison of TraH_57–183_ to related proteins. (**a**) Secondary structure (PSIpred) and TM motif prediction (HMMTOP) for G- and G+ VirB8-like proteins with emphasis on the identified G+ class ALPHA candidates; α-helices (red), β-strands (blue), and TM motifs (boxed) are highlighted. Methods for experimentally determined secondary structures are indicated where applicable. (**b**) Cartoon representation of NTF2 (1OUN), TraH_57–183_ (5AIW), VirB8_At_ (2CC3), TraM_214–322_ (4EC6), TcpC central (residues 104–231) and C-terminal (residues 232–354) domain (3UB1), DotI_Lg_ (3WZ4).

In contrast, the C-terminal VirB8-like domain of TraM crystallized as a trimer, presumably facilitated by a putative triple coiled-coil motif close to the TM helix^27^. The triple coiled-coil feature was also detected in G+ TcpC from *C. perfringens,* which serves, due to two VirB8 domains, as prototype for the BETA class of VirB8 molecules. Furthermore, deletion derivatives of TcpC tested in bacterial two-hybrid analysis suggested that, amongst others, the first 98 N-terminal residues and in particular the TM domain is essential for homo-oligomerization and for the interaction with TcpA, TcpG and TcpH^33^. Likewise, oligomerization of TraH might be promoted by a similar mechanism as well. In fact, a putative dimeric coiled-coil motif was predicted with high probability between residues Glu33-Glu55^57^, a motif that appears to be absent in all tested G- VirB8 members (Supplementary Fig. S3). Nevertheless, membrane localization and/or additional contacts provided by the N-terminal TM helix seem to be required for oligomerization as shown by SEC experiments, chemical *in vitro* as well as *in vivo* crosslinking and semi-native PAGE analysis. While in TraH this putative coiled-coil is predicted as the first secondary structure element after the TM helix, the TM helix is followed by two predicted short β-strands in G- VirB8-like class ALPHA proteins. These differences mark TraH as being structurally distinct from the classical G- class ALPHA proteins. Interestingly, TraH shares the partly distinct structural composition with the previously predicted G+ class ALPHA members, but is even shorter^27^ (Fig. 7a).

It is surprising that TraH only shows a very limited number of de-facto identical structural relatives in a broad spectrum of conjugative plasmids, transposons, ICEs and GIs from G+ bacteria. Only some of the known enterococcal G+ T4SSs encode a putative transfer protein with a VirB8 class-ALPHA secondary structure composition. This suggests an exclusive role for TraH-like proteins in the respective T4SSs, which is in contrast to the high incidence of relaxases, coupling proteins, transglycosylases, ATPases in virtually all conjugative T4SSs in general and other VirB8-like protein classes in particular^27^.

It seems that the original G- class ALPHA VirB8 like proteins have been structurally adapted to fit the different needs of G+ T4SSs for example by gene duplication (in case of the two VirB8-like domains of TcpC from pCW3) or mutation, giving rise to the other two prevalent classes. Only in a hand full of T4SSs the classical composition of the class ALPHA VirB8-like proteins has been largely preserved. It is likely that these secondary proteins are key for the adaption of the respective T4SS-machinery to their G+ hosts. The *traH* knock-out mutant clearly demonstrated that the protein is such an essential component for pIP501-mediated conjugation in G+ bacteria. Transfer rates of pIP501Δ*traH* dropped by three orders of magnitude compared to wt pIP501. Full recovery of the transfer capacity when supplying *traH in trans* excluded polar effects of the deletion on downstream *tra* genes. Transfer frequencies of the complemented mutant were identical with and without induction of *traH* expression; this is in agreement with data from Eichenbaum *et al*.^58^ who observed that the pEU327 *xylA* promoter is constitutive in *E. faecalis*^58^. The indispensability of TraH suggests non-redundant roles of the two VirB8-like proteins TraH and TraM in the pIP501 T4SS. In fact, TraM was also found to be essential for pIP501-mediated conjugation in *E. faecalis* (E. Grohmann, personal communication, October 2015).

**Figure 8:**
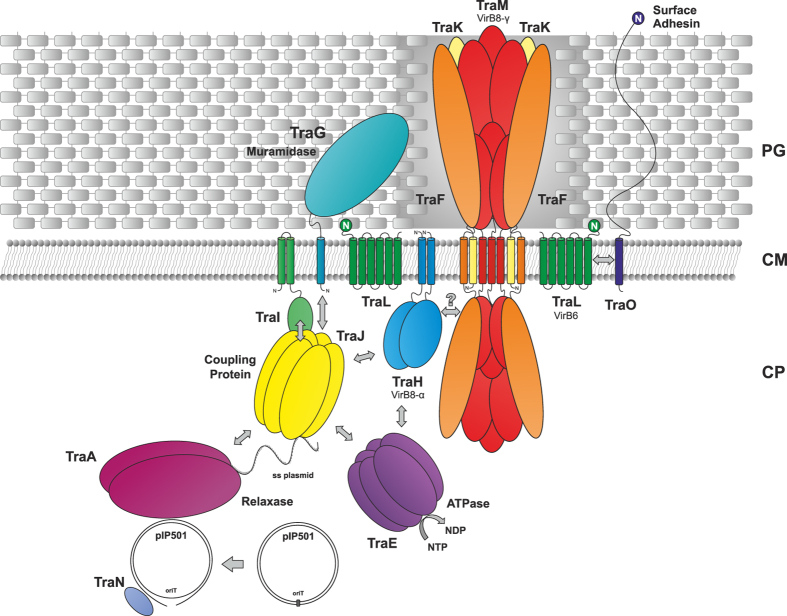
Localization and interaction model of the pIP501 encoded T4SS. The localization, orientation and interactions of depicted transfer proteins is based on *in silico* predictions and experimental data^13^,^16^,^25^,^26^,^27^,^28^. Protein-protein interactions are either indicated by arrows or overlapping protein surfaces. Presence of TraF, TraK and TraM in the core complex of the system is inferred from homology in case of TraM and further based on preliminary interaction data found by the bacterial two-hybrid assay (Kohler *et al*., unpublished data). As no interaction of TraH with putative core complex proteins could be detected so far participation of TraH herein remains speculative and is therefore marked with a question mark. The putative function of pIP501 key proteins and the VirB8 class-affiliation of TraH and TraM are specified. PG, peptidoglycan; CM, cytoplasmic membrane; CP, cytoplasm.

Based on the data presented here and the striking structural similarities between TraH and G- VirB8-like proteins (Fig. 7b) we further refined our previously proposed model for the pIP501 conjugative machinery^59^. Considering the reversed topology of TraH with the VirB8-like domain pointing towards the inside of the cell, we suggest a function as scaffolding or assembly/recruitment factor in a membrane embedded complex at the cytoplasmic site of the G+ membrane (Fig. 8). In contrast, the C-terminal VirB8-like domain of TraM might fulfill a similar task at the extracellular site of the membrane. Moreover, it was suggested that TraM could also act as attachment site for the recipient cell during conjugative transfer due to its surface accessibility^27^. Similarly, the C-terminal VirB8 domain of TcpC was found to be ideally positioned in the respective crystal structure to serve as major interaction surface for other pCW3 transfer proteins. Bacterial two-hybrid analysis further confirmed its vital role for the interaction with TcpA, TcpG and TcpH^33^. Considering a TcpC topology with the VirB8 double-domain located outside the cell, TraM and TcpC might share a common function in their respective T4SSs. As TraH represents the first class ALPHA VirB8-like protein to be structurally confirmed in a G+ T4SS and there is still no experimental interaction data available, we can currently only speculate about its definite role in the pIP501 conjugative conduit. In agreement with its proposed role as nucleation and assembly factor for other channel components in the cytoplasm, association of TraH with these factors is likely to be transient and relatively weak. In case of G- bacteria, ALPHA class VirB8 proteins were found to interact with VirB3, VirB5, VirB6, VirB9 and VirB10 in mutational analysis and binding studies^51^,^54^, ELISA^53^ and crosslinking, pull-down and FRET-based experiments^60^. Furthermore, close contact of VirB8 from *A. tumefaciens* to the transferred DNA strand (T-DNA) was detected by a quantifiable transfer DNA immunoprecipitation assay (TrIP)^5^. So far, TraH was only found to interact with the double-strand DNA binding protein TraN in yeast two-hybrid and pull-down studies^13^. However, NMR titration experiments with purified TraH_53–183_ and TraN failed to confirm these findings (data not shown). Interestingly, preliminary interaction data applying a fluorescence-based thermal shift assay suggested weak binding of TraH to the VirB4-like ATPase TraE and the VirD4-like coupling protein TraJ^16^. Hence, a putative role of TraH might be the recruitment of these essential T4SS factors to the assembled channel complex.

Despite the growing functional and structural information on T4SSs in general and G+ conjugation in particular, further efforts are needed to fully uncover the function of VirB8-like proteins. We propose that the presence of a second VirB8-like protein in the pIP501 transfer complex will help in elucidating structure-function relationships of this protein family in T4SSs by providing novel insights in the functional and structural adaptation among conjugative transfer systems.

## Materials and Methods

### Subcellular fractionation of *E. faecalis* JH2-2 (pIP501) and immunolocalization of TraH

Subcellular fractionation of *E. faecalis* JH2-2 (pIP501) was performed as described in Goessweiner-Mohr *et al*.^26^. TraH was probed in the fractions (cell wall, membrane, cytoplasm) by immunostaining with primary rabbit polyclonal TraH-specific antibodies (Biogenes, Berlin, Germany) and a secondary horseradish peroxidase conjugated antibody against rabbit IgG (Promega GmbH, Mannheim, Germany).

### Circular-dichroism spectroscopy

CD measurements were performed on a Jasco J715 spectro-polarimeter (Jasco, Groß-Umstadt, Germany) connected to an external thermostat. Spectra were recorded from 260 to 190 nm in a 0.02 cm cuvette. The average spectrum and the standard deviation of ten individual measurements were calculated. Protein stock solutions of TraH_29–183_ and TraH_57–183_ were diluted to 0.8 mg/ml for all experiments. CD spectra were normalized and evaluated using the online service Dichroweb (http://dichroweb.cryst.bbk.ac.uk) employing the CDSSTR algorithm and reference data set #4^61^.

### Analytical size exclusion chromatography (SEC)

For analytical purposes, 500 μl solution containing 0.5 mg/ml purified TraH_29–183_ and TraH_57–183_, respectively, were applied on a pre-packed Superdex 200 HR 10/30 column (GE Healthcare) with a constant flow rate of 0.5 ml/min. A gel filtration standard (670/158/44/17/1.35 kDa, BioRad, Hercules, CA) was used to calculate the molecular weight of the eluting fractions.

### Small angle X-ray scattering (SAXS) experiments

All samples subjected to SAXS experiments were first analyzed for mono-dispersity employing dynamic light scattering (DLS - DynaPro MSXTC device, Protein Solutions, Chicago, IL) up to a protein concentration of 10 mg/ml (Supplementary Fig. S2). SAXS experiments were conducted at beamline X33^62^ at DESY Hamburg equipped with a Pilatus 1M detector at TraH_29–183_ concentrations of 2.8 and 5.3 mg/ml, respectively. To prevent radiation damage, buffer A was supplemented with 5% (v/v) glycerol and 1 mM DTT and used during all SAXS measurements. The detector-sample distance was set to 2.7 m covering a range of momentum transfer (s) of 0.06–6 nm^−1^ at an X-ray wavelength of 0.15 nm.

### Data analysis and low resolution shape reconstruction

Initial buffer subtraction, scaling and merging of the individual scattering data was performed using the program PRIMUS^63^. Maximum intensity (I_0_) and the radius of gyration (R_g_) were calculated from the Guinier plot. The maximum diameter (D_max_) of the particle was set to 6.5 nm and the pair distance distribution function was generated with GNOM^64^. From the generated output file 20 independent *ab initio* models were reconstructed in reciprocal space using GASBOR22I^65^. 19 models of this initial set were further processed and averaged with the programs SUPCOMB^66^ and DAMAVER^67^. The theoretical scattering curve of each TraH_57–183_ NMR model was calculated and fitted to the experimental scattering curve of TraH_29–183_ using the program CRYSOL^36^.

### Protease protection assay

Protease protection assays were performed as described by Goessweiner-Mohr *et al*.^26^. Briefly, enzymatically produced protoplasts were treated with increasing amounts of proteinase K before complete degradation by Triton X-100. Samples were loaded on SDS-polyacrylamide gels and subjected to western blotting. Blots were probed with rabbit polyclonal TraH-specific antibodies (BioGenes) followed by a secondary horseradish peroxidase conjugated antibody directed against rabbit IgG (Promega GmbH). Immuno detection of the extracellular, membrane anchored transfer protein TraK and the cytoplasmic protein TraN in the same sample served as controls for extra-and intracellular proteins, respectively.

### Chemical crosslinking

For the *in vitro* crosslinking studies of the purified proteins, a total amount of 8 μg protein were incubated in 300 mM NaCl, 100 mM bicine, 1 mM DTT with increasing amounts of freshly prepared glutaraldehyde (GA) solution (0–1%, v/v) for 15 min at RT. Full-length TraH was kept reconstituted in DDM micelles during the course of the experiment to avoid unspecific aggregation of the protein. Reactions were stopped by the addition of glycine to a final concentration of 140 mM. Proteins were acetone-precipitated at −20 °C overnight and suspended in 20 μl SDS-PAGE loading buffer.

*In vivo* FA crosslinking was carried out as previously described^68^. In brief, *E. faecalis* JH2-2 cells (pEU327-*RBS-traH*)^58^ were harvested in early log phase and treated for 10 minutes with FA in PBS (0.5–2%). After inactivation with 1.25 M glycine, cells were lysed by sonication, suspended in SDS-PAGE buffer and incubated for 20 minutes at 60 °C. Gel electrophoresis was performed in 12.5% SDS-PAGE gels. TraH was probed by western blotting as described for the protease protection assay.

### Semi-native PAGE analysis

The semi-native PAGE was carried out as previously described^69^ with some minor modifications. In brief, purified TraH and TraH_29–183_ (0.5 and 1 μg, respectively) were mixed with native PAGE sample buffer without SDS and reducing agent and kept at 4 °C. Purified full-length TraH was kept reconstituted in DDM micelles during the course of the experiment. Samples were loaded onto 12.5% native polyacrylamide gels and electrophoresis was performed at 100 V and 4 °C in common SDS-PAGE running buffer containing 0.1% SDS for 2.5–4 hours. TraH was probed by western blotting as described for the protease protection assay.

### NMR experiments

NMR measurements were carried out on a Bruker Avance III 700 MHz spectrometer equipped with a cryogenically cooled TCI probe head. All experiments were recorded at 298 K using a 1 mM uniformly ^15^N/^13^C-labeled sample. Spectra were processed with NMRPipe^70^ and analyzed with Sparky^31^. Resonance assignment was performed as described elsewhere^71^. Distance information was obtained from ^15^N- and ^13^C-edited NOESY spectra with a mixing time of 70 ms^72^. ^15^N R_1_, R_2_-relaxation rates and {^1^H}-^15^N heteronuclear NOE data were measured at a 700 MHz proton Larmor frequency as described^73^.

### Structure calculation

CYANA 3.0 was used for automated NOE cross-peak assignment^74^. Distance restraints from the CYANA calculation- and TALOS+ derived torsion angles^75^ were used in a water refinement calculation^76^ using the RECOORD protocol^77^. The quality of the structure ensemble was validated using the iCING web server^78^. Molecular images were generated using PyMOL (Delano Scientific, Palo Alto, CA) and the UCSF Chimera package^79^.

### Sequence based comparison and characterization

The search for other TraH-like proteins in G+ conjugative plasmids, transposons, integrative conjugative elements (ICEs), and genetic islands (GIs) was performed by comparing secondary structure elements predicted by PSIpred^80^ and the position of TM helices identified by HMMTOP^81^ to the known structures of VirB8, TcpC, TraM and TraH.

### Referenced accessions

PDB-codes: 1OUN, 2CC3, 2BHM, 4KZ1, 4NHF, 3UB1, 4EC6, 3WZ4.

## Additional Information

**Accession codes**: Coordinates and structure factors for TraH57–183 have been deposited in the Protein Data Bank under accession code 5AIW.

**How to cite this article**: Fercher, C. *et al*. VirB8-like protein TraH is crucial for DNA transfer in *Enterococcus faecalis. Sci. Rep.* **6**, 24643; doi: 10.1038/srep24643 (2016).

## Supplementary Material

Supplementary Information

Supplementary Video S1
