# Supplementary material for: VirB8-like protein TraH is crucial for DNA transfer in Enterococcus faecalis
Source: Sci Rep. 2016 Apr 22;6:24643. doi: 10.1038/srep24643 (PMC4840375; doi:10.1038/srep24643)
Supplement: Supplementary Information [file srep24643-s1.pdf]

Supplemental information

VirB8-like protein TraH is crucial for DNA transfer in *Enterococcus faecalis*

Christian Fercher[1,9], Ines Probst[2,3], Verena Kohler[1], Nikolaus Goessweiner-Mohr[4], Karsten Arends[5], Elisabeth Grohmann[2,6], Klaus Zangger[7], N. Helge Meyer⌧[8,9], Walter Keller⌧[1]

[1]Institute of Molecular Biosciences, NAWI Graz, University of Graz, Austria.
[2]Division of Infectious Diseases, University Medical Center Freiburg, Germany.
[3]Faculty of Biology, Microbiology, Albert-Ludwigs-University Freiburg, Germany.
[4]1] Center for Structural System Biology (CSSB), University Medical Center Hamburg-Eppendorf (UKE), Hamburg, Germany; 2] Deutsches Elektronen-Synchrotron (DESY), Hamburg, Germany; 3] Institute of Molecular Biotechnology (IMBA), Austrian Academy of Sciences, Vienna, Austria; 4] Research Institute of Molecular Pathology (IMP), Vienna, Austria.
[5]Robert Koch Institute, Berlin, Germany.
[6]Beuth University of Applied Sciences, Berlin, Germany.
[7]Institute of Chemistry, University of Graz, Graz, Austria.
[8]Department of General and Visceral Surgery, University of Oldenburg, Germany.
[9]These authors contributed equally to this work.

⌧Correspondence should be addressed to N.H.M. (helge.meyer@uni-oldenburg.de) or W.K. (walter.keller@uni-graz.at).

Supplemental methods

Strains and growth conditions

Bacterial strains used in this work are listed in **Supplementary Table S4**. All *E. faecalis* strains were grown in brain heart infusion (BHI) medium (Oxoid, Hampshire, United Kingdom) at 37°C, supplemented with respective antibiotics, when required; *E. faecalis* JH2-2 (pIP501) and *E. faecalis* JH2-2 (pIP501Δ*traH*) supplemented with 50 µg/ml fusidic acid (Fus), 20 µg/ml erythromycin (Em), 20 µg/ml chloramphenicol (Cm), *E. faecalis* JH2-2 (pIP501::pKAΔ*traH*) with 50 µg/ml Fus, 20 µg/ml Em, 20 µg/ml Cm and 150 µg/ml gentamicin (Gent) and *E. faecalis* JH2-2 (pIP501Δ*traH*, pEU327-RBS-*traH*) with 50 µg/ml Fus, 20 µg/ml Em, 20 µg/ml Cm and 500 µg/ml spectinomycin (Spec) and *E. faecalis* OG1X supplemented with 1.5 mg/ml streptomycin (Sm). *E. coli* EC1000 (pKA) was cultured in BHI with 50 µg/ml kanamycin (Km), 20 µg/ml Gent, 100 µg/ml Em and *E. coli* EC1000 (pKAΔ*traH*) in BHI with 50 µg/ml Km, 20 µg/ml Gent and 250 µg/ml Em. *E. coli* DH5α harboring pUC18 or derivatives thereof in Luria-Bertani (LB) medium with 100 µg/ml ampicillin (Amp)*, E. coli* DH5α (pEU327) and *E. coli* DH5α (pEU327-RBS-*traH*) in LB medium with 100 µg/ml Spec.

Construction of a *traH* in-frame knockout mutant

A pIP501 *traH* in-frame deletion mutant was generated using a method consisting of two homologous recombination steps as described in Arends et al. (2013) with minor modifications[1]. We deleted 93 % of *traH* maintaining the 6 N-terminal and the 6 C-terminal codons intact to avoid polar effects on downstream genes in the pIP501 *tra* operon. Oligonucleotide primers and restriction enzymes are listed in **Supplementary Table S5**.

First, the pIP501 *traH* up- and downstream (1014-bp and 970-bp) regions were amplified by PCR, inserted into pUC18 and transformed into *E. coli* DH5α cells. Clones were selected on LB agar plates supplemented with 100 µg/ml Amp. To obtain pKAΔ*traH*, the fused up- and downstream regions of pUC18-UPS-DWS-*traH* were cut with *Pst*I/*Eco*RI, inserted into *Pst*I/*Eco*RI-cut pKA and transformed into *E. coli* EC1000 followed by selection on BHI agar supplemented with 250 µg/ml Em. *E. faecalis* JH2-2 (pIP501) was electroporated with the suicide vector pKAΔ*traH*, transformants were selected on

BHI agar supplemented with 50 µg/ml Fus, 20 µg/ml Em, 20 µg/ml Cm, 100 µg/ml Gent and 100 µl of a 20 mg/ml 5-bromo-4-chloro-3-indolyl-β-D-galactopyranoside(X-gal) solution. *E. faecalis* JH2-2 (pIP501) colonies were screened for integration of pKAΔ*traH* at homologous sites into pIP501 by PCR. Colonies with integrated pKAΔ*traH* were grown in BHI medium supplemented with 50 µg/ml Fus, 20 µg/ml Cm three times to stationary phase and finally until an OD_{600} of 0.4. Serial dilutions were spread on MM9YEG agar supplemented with 50 µg/ml Fus, 20 µg/ml Cm, 15 mM DL-p-chlorophenylalanine and 100 µl of a 20 mg/ml X-gal solution. Colonies were screened for *traH* in-frame deletion by PCR. *E. faecalis* JH2-2 pIP501Δ*traH* mutants were verified by sequencing of the deletion borders.

Complementation of *E. faecalis* (pIP501Δ*traH*)

The *traH* wild type gene cloned into the expression vector pEU327[2] was used to complement the markerless *traH* deletion *in trans*. *traH* including its ribosomal binding site (RBS) was amplified from pIP501 with primers listed in **Supplementary Table S5**. The 600-bp RBS-*traH* fragment was inserted into plasmid pEU327 and transformed into *E. coli* DH5α cells. Clones were selected with 100 µg Spec/ml and tested for insertion of RBS-*traH* by PCR and sequencing. pEU327-RBS-*traH* was electroporated into *E. faecalis* JH2-2 (pIP501Δ*traH*) to complement the deletion mutant.

Biparental matings

BHI medium supplemented with required antibiotics was inoculated with overnight cultures of the respective donor and recipient strains and incubated until an OD_{600} of 0.5. Donor and recipient cultures were washed twice in 1 ml BHI medium and suspended in 1 ml phosphate-buffered saline (PBS). Serial dilutions of *E. faecalis* OG1X were plated on BHI agar supplemented with 1.5 mg/ml Sm to enumerate recipients. *E. faecalis* JH2-2 (pIP501) or *E. faecalis* JH2-2 (pIP501Δ*traH*) donors were mixed in a volume ratio 1:9 with recipient *E. faecalis* OG1X. Mating mixtures were centrifuged for 1 min at 6000 x g, cell pellets were suspended in 30 µl PBS and spotted onto a BHI agar plate. Plates were incubated overnight at 37°C; cells were recovered with 1 ml PBS and plated on BHI agar supplemented with 1.5 mg/ml Sm and 20 µg/ml Em to select for transconjugants. To test

complementation of pIP501Δ*traH* with wt (wild type) *traH* supplied *in trans*, matings with *E. faecalis* JH2-2 (pIP501Δ*traH*; pEU327-RBS-*traH*) as donor and *E. faecalis* OG1X as recipient were performed. All matings were carried out in triplicates and both recipients and transconjugants were enumerated after 24h. Mean transfer rates (transconjugants per recipient cell) and standard deviations are given.

Cloning, expression and purification of native TraH variants

The DNA sequences coding for full length TraH, $TraH_{29-183}$ and $TraH_{57-183}$, were amplified from pIP501 by PCR using Q5 polymerase (New England Biolabs, Ipswich, MA) and primers containing restriction sites for subsequent cloning into the expression vector pQTEV (kindly donated by K. Büssow, Max-Planck-Institute for Molecular Genetics, Berlin).

E. coli BL21 (DE3) CodonPlus® cells (Stratagene, La Jolla, CA) were transformed with recombinant pQTEV-*traH* and *traH* truncation variants. Cells were grown at 37°C in selective LB medium containing 100 µg/ml Amp to an OD_{600} of 0.7. Cultures were chilled at 4°C for 10 min and protein expression was induced by adding isopropyl-β-D-thiogalactopyranoside (IPTG) to a final concentration of 0.5 mM. Protein expression continued for 18 h at 16°C. Cells were harvested and suspended in buffer A (50 mM Bis-Tris, pH 6.2, 100 mM NaCl, 100 mM $(NH_4)_2SO_4$, 1 mM benzamidine, 0.1 mM phenylmethanesulfonyl fluoride (PMSF). The suspension was homogenized (UltraTurrax, IKA, Staufen, Germany) and bacterial cells were disrupted by sonication (Sonopuls HD2070, Bandelin, Berlin, Germany; 5 min, discontinuous sonication, 70 % amplitude). After 30 min at 4°C, cell debris and insoluble material were separated from the soluble fraction by centrifugation for 30 min at 4 °C and 30,000 × g.

The supernatant containing the His_7-tagged protein was purified by immobilized metal ion affinity chromatography (IMAC) on a 1 ml HisTrap FF column (GE Healthcare, Chalfont St. Giles, United Kingdom). Protein was eluted in 15 column volumes (CV) with a linear imidazole gradient ranging from 50-250 mM. Fractions of 1 ml were collected and protein yield and purity were assessed by SDS-PAGE. TraH containing fractions were pooled and cleaved overnight at 4°C with TEV protease in a ratio of 1 mg TEV per 100 mg protein, additionally supplying 1 mM EDTA and 1 mM dithiothreitol

(DTT). Simultaneously, imidazole was removed by dialysis using a membrane with a molecular weight cutoff (MWCO) of 3,500 Da. TEV protease, uncleaved protein and cleaved His-tag were removed in a second IMAC step. TraH was further purified by preparative size exclusion chromatography on a Superdex 200 10/30 column (GE Healthcare) equilibrated with 50 mM Bis-Tris, pH 6.2, 100 mM NaCl, 100 mM $(NH_4)_2SO_4$. Finally, the purified protein was concentrated by ultrafiltration (Amicon concentration tubes, 3,000 Da MWCO). Purity was assessed via SDS-PAGE and the protein concentration was measured via the absorbance at 280 nm using a NanoDrop® ND-1000 spectrophotometer (PEQLAB Biotechnologie GmbH, Erlangen, Germany). Isotopically labeled TraH variants were expressed and purified as described elsewhere[3]

Full length TraH was expressed and extracted from the bacterial culture analog to $TraH_{29\text{-}183}$ and $TraH_{57\text{-}183}$ with minor modifications. Prior to IMAC purification, TraH was extracted from the insoluble fraction using phosphate buffered saline (PBS) supplemented with 1% (v/v) Triton X-100 and 0.5% (w/v) *n*-dodecyl β-D-maltoside (DDM). Before elution of the protein detergent concentration was reduced to 0.1% (w/v) DDM. The purity of individual fractions was assessed by SDS-PAGE and TraH containing fractions were pooled and dialyzed overnight against PBS at 4°C.

Supplemental tables

Supplementary Table S1: Refinement statistics for TraH

**Distance constraints**	
Total NOEs	3617
Intra-residue	840
Inter-residue	2777
Sequential ($\|i - j\| = 1$)	972
Medium-range ($\|i - j\| < 4$)	657
Long-range ($\|i - j\| > 5$)	1148
Intermolecular	0
Hydrogen bonds	0
Total dihedral angle restraints	97
ϕ	94
ψ	97
**Structure statistics**	
**Violations (mean and s.d.)**	
Distance constraints (Å)	0.013 ± 0.001
Dihedral angle constraints (°)	0.433 ± 0.075
Max. dihedral angle violation (°)	3.88
Max. distance constraint violation (Å)	0.30
**Deviations from idealized geometry**	
Bond lengths (Å)	0.014
Bond angles (°)	1.500
Impropers (°)	1.538
**Average pairwise r.m.s. deviation[a] (Å)**	
Heavy	0.89 ± 0.12
Backbone	0.51 ± 0.11

[a]Pairwise r.m.s. deviation was calculated among 20 refined structures for residues 60-177

Supplementary Table S2: Structural alignments with DALI (A) and MATRAS (B)[4,5]

Entry number	PDB code	Z-score	RMSD [Å] (A) / Rdis [%] (B)	Sequence identity [%]	Aligned residues	Description
**A-1**	2bhm	11.2	2.6	10	110	T4SS protein VirB8 from *Brucella suis*
**A-2**	4nhf	11.1	2.8	14	116	T4SS protein TrwG from *Bartonella grahamii*
**A-3**	4o3v	10.9	2.7	19	112	T4SS protein VirB8 from *Rickettsia typhi*
**A-4**	3wz3	10.9	2.6	10	114	T4SS protein TraM from plasmid R64
**A-5**	4lso	10.9	2.6	6	113	T4SS protein VirB8 from *Bartonella quintana*
**A-6**	4jf8	10.7	2.8	14	115	T4SS protein TrwG from *Bartonella birtlesii*
**A-7**	2cc3	10.5	2.6	10	112	T4SS protein VirB8 from *Agrobacterium tumefaciens*
**A-8**	3wz4	10.4	2.8	8	118	T4SS protein DotI from *Legionella pneumophila*

**A-16**	3ub1	9.2	3.2	14	106	T4SS protein TcpC from *Clostridium perfringens* (pCW3)
**A-36**	4ec6	8.5	3.0	7	99	T4SS protein TraM from *Enterococcus faecalis* (pIP501)
**B-1**	4jf8	28.27	40.3	13	91-230	T4SS protein TrwG from *Bartonella birtlesii*
**B-2**	4lso	27.51	40.3	8	95-234	T4SS protein VirB8 from *Bartonella quintana*
**B-3**	2cc3	26.89	38.7	9	91-228	T4SS protein VirB8 from *Agrobacterium tumefaciens*
**B-4**	4o3v	26.74	40.8	17	90-229	T4SS protein VirB8 from *Rickettsia typhi*
**B-5**	3ub1	26.27	19.7	14	105-233	T4SS protein TcpC from *Clostridium perfringens* (pCW3)
**B-6**	3b8l	25.70	35.4	8	4-136	Putative aromatic ring hydrolase from *Novosphingobium aromaticivorans*
**B-7**	1idp	25.41	34.7	5	12-153	Scytalone dehydratase F162A mutant
**B-8**	1tuh	25.38	37.8	10	20-142	Bal32a (alpha-beta-barrel family)
**B-9**	3a76	24.72	24.72	5	6-137	Gamma-hexachlorocyclohexane dehydrochlorinase LinA
**B-77**	4ec6	15.55	33.7	9	214-322	T4SS protein TraM from *Enterococcus faecalis* (pIP501)

Supplementary Table S3: Transfer rates (mean value and standard deviation of three independent assays)

**Donor**	**Recipient**	**Transfer rate [transconjugants/recipient]**
E. faecalis JH2-2 (pIP501)	*E. faecalis* OG1X	$2.3 \times 10^{-5} \pm 2.5 \times 10^{-7}$
E. faecalis JH2-2 (pIP501) merodiploid-derived	*E. faecalis* OG1X	$2.2 \times 10^{-5} \pm 1.5 \times 10^{-6}$
E. faecalis JH2-2 (pIP501Δ*traH*)	*E. faecalis* OG1X	[a]$< 2.3 \times 10^{-8} \pm 7.9 \times 10^{-10}$
E. faecalis JH2-2 (pIP501Δ*traH*, pEU327-RBS-*traH*)	*E. faecalis* OG1X	$2.9 \times 10^{-5} \pm 1.9 \times 10^{-6}$
E. faecalis JH2-2 (pIP501Δ*traH*, pEU327-RBS-*traH* induced)	*E. faecalis* OG1X	$3.0 \times 10^{-5} \pm 8.3 \times 10^{-6}$

[a] transfer rate was below the detection limit of the assay

Supplementary Table S4: Bacterial strains and plasmids used in this work

Strain or plasmid	Genotype or description[a]	Reference or source
**Strains**		
E. coli DH5α	F- ϕ80*lacZ*ΔM15 Δ(*lacZ YA-argF*)*U169 recA1 endA1 hsdR17*(rK- mK+) *phoA supE44 thi-1 gyrA96 relA1* λ-	Invitrogen
E. coli EC1000	F- RepA+ *araD139* (*araABC-leu*)*7679 galU galK lacX74 rspL thi* Km^r	[6]
E. coli BL21-CodonPlus(DE3)-RIL	F_ *ompT hsdS(rB-mB-) dcm-* Tet^r *gal -* (DE3) *endA Hte [argU ileY leuW* Cm^r]	Stratagene
E. faecalis JH2-2	Rif^r Fus^r	[7]
E. faecalis OG1X	Sm^r	[8]
**Plasmid**		
pIP501	*tra*+ Cm^r MLS^r	[9]
pUC18	Amp^r	Promega
pUC18-UPS-*traH*	pUC18 with *traH* upstream regions at *PstI/XbaI* sites	This study
pUC18-UPS-DWS-*traH*	pUC18-UPS-*traH* with *traH* downstream regions at *BamHI/EcoRI* sites	This study
pKA	pCJK47 *aacA-aphD* at *Bgl*II site; Em^r Gent^r	[1]
pKAΔ*traH*	pKA with *traH* up- and downstream regions at *Pst*I/*Eco*RI sites	This study
pIP501Δ*traH*	pIP501 *traH* in-frame deletion	This study
pEU327	*E. coli*/G+ bacteria shuttle plasmid, Spec^r *xylA* promoter	[2]
pEU327-RBS-*traH*	pEU327 with RBS-*traH*	This study
pQTEV	P_{t4} *lacI*^{q} His_7 Amp^r	[10]

[a] Cm^r, chloramphenicol resistance; Km^r, kanamycin resistance; Fus^r, fusidic acid resistance; Spec^r, spectinomycin resistance; Sm^r, streptomycin resistance; Amp^r, ampicillin resistance; Gent^r, gentamicin resistance; MLS^r, macrolide-lincosamide-streptogramin B resistance; *tra*+, transfer proficient.

Supplementary Table S5: Oligonucleotides used in this work

Primer	Sequence (5'- 3')[a]	Position / Reference
***traH* in frame deletion**		
*Pst*I_UPS_*traH* fw	GCG**CTGCAG**AGTGGCGGACTAGCAAC	169-185[c]
*Xba*I_UPS_*traH* rev	GGC**TCTAGA**TATTTTGTCAAACTCCATTTC	1162-1182[c]
*Bam*HI_DWS_*traH* fw	GCC**GGATCC**CAATTAGGAGAGGAGTAGA	1699-1717[c]
*Eco*RI_DWS_*traH* rev	CTC**GAATTC**TCTTCGTTATAGCGTGGAT	2650-2668[c]
**Screening in frame deletion**		
delta_*traH* _A fw	GAGTATCTGGTTCAAACTA	7804-7822[b]
delta_*traH* _B rev	TGTTTGGTCTTGCTTCAC	2761-2778[c]
delta_*traH* _C fw	AGGAAGAAATGGAGTTTGA	1157-1175[c]
delta_*traH* _D rev	CTTCGCCATGCTTCTAC	1713-1729[c]
***traH* complementation**		
pEU327_*Sal*I_RBS_*traH* fw	CGC**GTCGAC**GCAAATATTGCAAGTTATGT	1126-1145[c]
pEU327_*Sal*I_RBS_*traH* rev	GGC**GTCGAC**GCCATGCTTCTACTCCTC	1708-1728[c]
**TraH expression**		
pQTEV_*traH* fw	GCG**GTCGAC**GGAAGAAATGGAGTTTGA	1158-1175[c]
pQTEV_*traH* rev	GGC**GCGGCCGC**TTCTACTCCTCTCCTA	1703-1718[c]
pQTEV_$traH_{29\text{-}183}$ fw	GCAC**GGATCC**ATTAAACAAGGCGAAC	1249-1264[c]
pQTEV_$traH_{29\text{-}183}$ rev	GCTT**GCGGCCGC**CTACTCCTCTCCTAATTG	1699-1716[c]
pQTEV_$traH_{57\text{-}183}$ fw	GCCC**GGATCC**AATACTAACCAAAGCGAATCG	1333-1353[c]
pQTEV_$traH_{57\text{-}183}$ rev	GCTT**GCGGCCGC**CTACTCCTCTCCTAATTG	1699-1716[c]
**pKA, pEU327 and pQTEV sequencing**		
pKA fw	GGAGACTACTTATTATGTAA	[11]
pKA rev	GCGCTTGTAATGTCATAT	[11]
pEU327 fw	CTTGCCAGTCACGTTACG	[2]
pEU327 rev	GATCAGCGATATCCACTTC	[2]
pQTEV fw	CCCGAAAAGTGCCACCTG	4712-4729[d]
pQTEV rev	GTTCTGAGGTCATTACTGG	277-295[d]

[a] added restriction sites are shown in **bold.**
[b] GenBank accession number L39769
[c] GenBank accession number NG_035648
[d] GenBank accession number AY243506

Supplemental figures

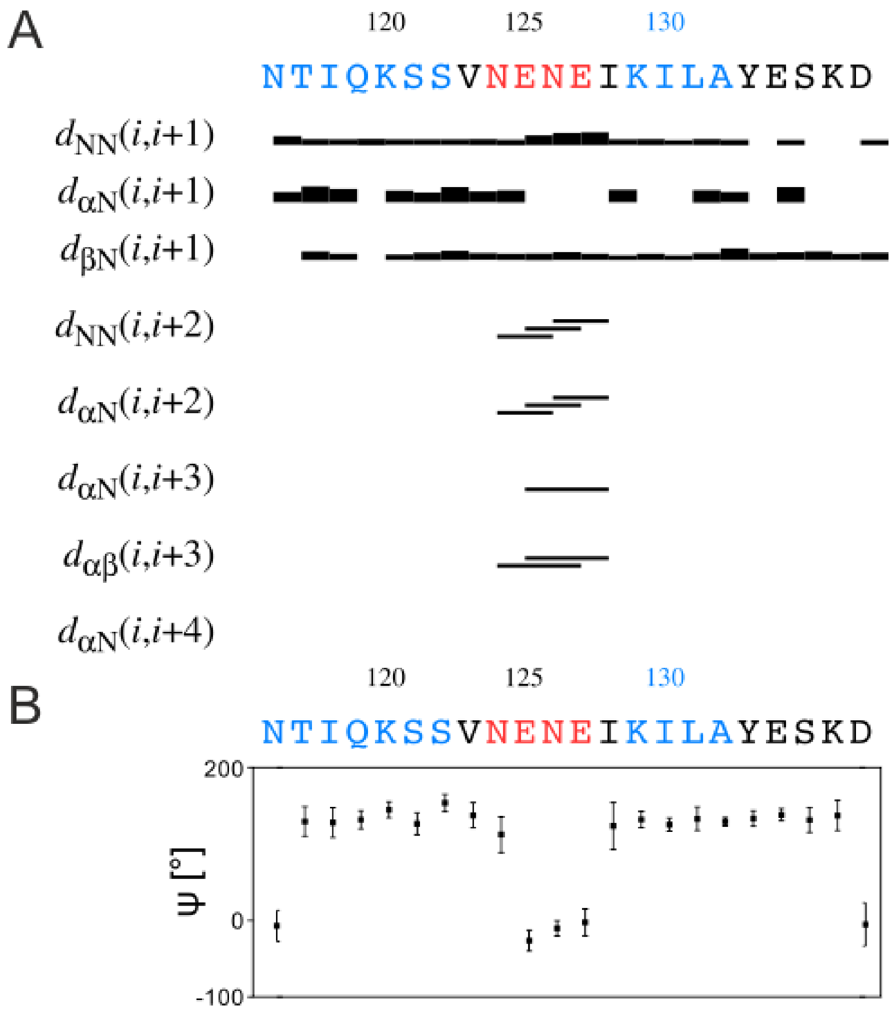

Fig. S1: NOE pattern (A) and TALOS+ derived dihedral angles (B) for residues within the distorted β-sheet β1. Sequence and residue numbers are indicated above.

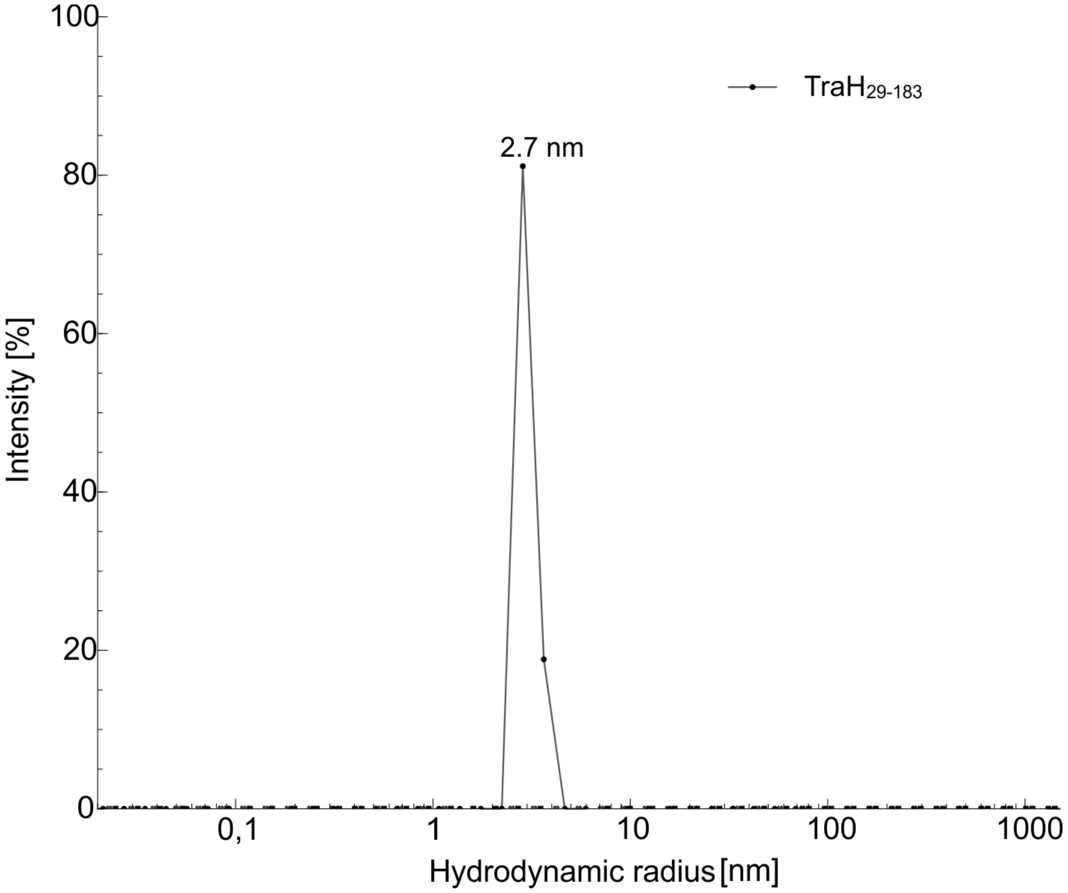

Fig. S2. DLS experiments of TraH$_{29\text{-}183}$. A narrow single peak is indicative of a homogenous, monodisperse species of particles in solution.

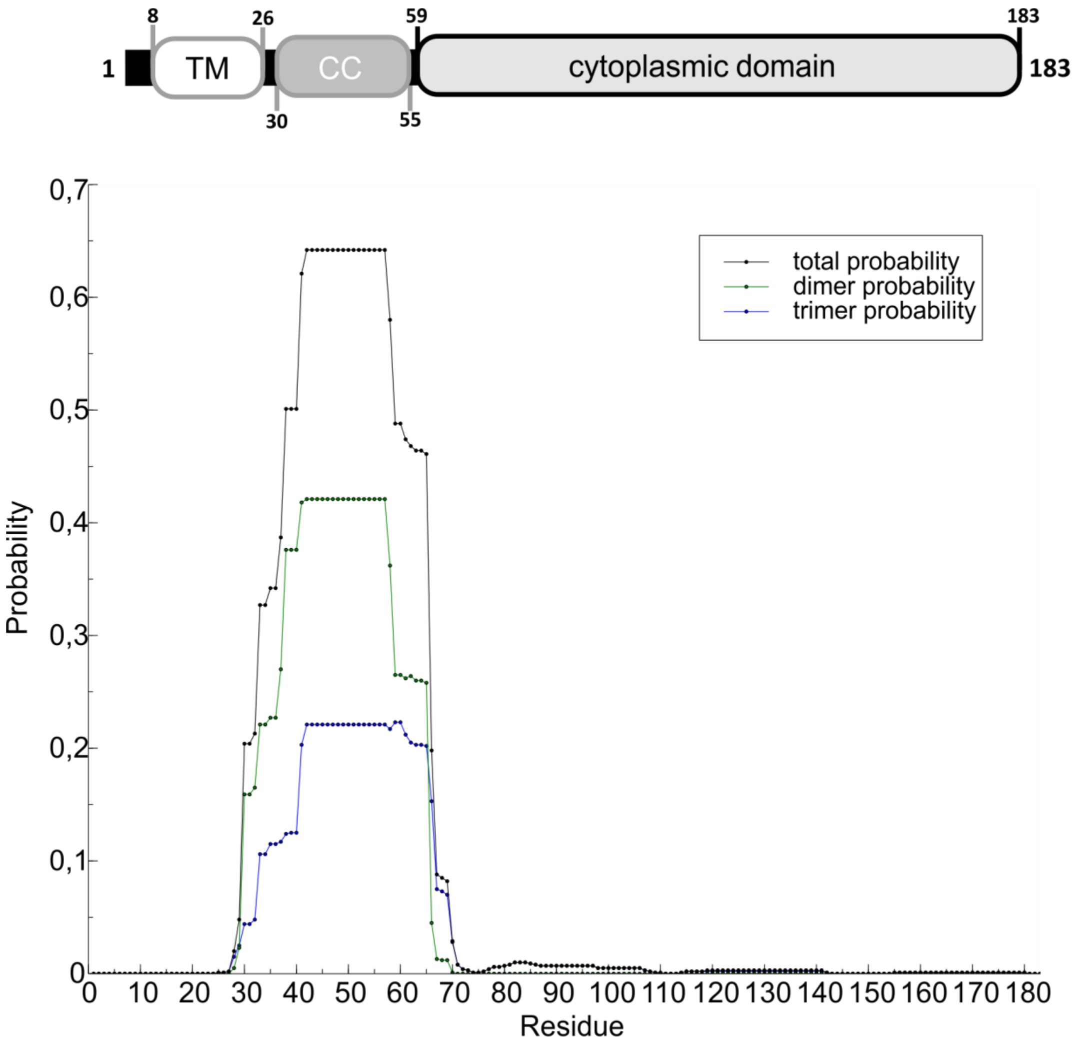

Fig. S3. Schematic view and analysis of TraH primary sequence towards the presence of coiled-coil (cc) motifs. According to the software Multicoil[12], there is a high score for a cc motif between residues Glu33-Glu55 with a higher probability for the dimeric state.

Supplemental video legend

Video S1. 360° view of the 20 lowest energy TraH$_{57\text{-}183}$ structures as calculated from NMR data. Residues contributing to the distorted β-sheet β1/ β1' and surface charge distribution are highlighted.

References cited in supplemental information

1. Arends, K. et al. TraG encoded by the pIP501 type IV secretion system is a two-domain peptidoglycan-degrading enzyme essential for conjugative transfer. *J Bacteriol* **195**, 4436-44 (2013).

2. Eichenbaum, Z. et al. Use of the *lactococcal* nisA promoter to regulate gene expression in gram-positive bacteria: comparison of induction level and promoter strength. *Appl Environ Microbiol* **64**, 2763-9 (1998).

3. Fercher, C., Keller, W., Zangger, K. & Meyer, N.H. ^{1}H, ^{15}N and ^{13}C chemical shift assignment of the Gram-positive conjugative transfer protein TraH. *Biomol NMR Assign* (2015).

4. Holm, L. & Rosenstrom, P. Dali server: conservation mapping in 3D. *Nucleic Acids Res* **38**, W545-9 (2010).

5. Kawabata, T. MATRAS: A program for protein 3D structure comparison. *Nucleic Acids Res* **31**, 3367-9 (2003).

6. Leenhouts, K. et al. A general system for generating unlabelled gene replacements in bacterial chromosomes. *Mol Gen Genet* **253**, 217-24 (1996).

7. Jacob, A.E. & Hobbs, S.J. Conjugal transfer of plasmid-borne multiple antibiotic resistance in *Streptococcus faecalis* var. zymogenes. *J Bacteriol* **117**, 360-72 (1974).

8. Ike, Y., Craig, R.A., White, B.A., Yagi, Y. & Clewell, D.B. Modification of *Streptococcus faecalis* sex pheromones after acquisition of plasmid DNA. *Proc Natl Acad Sci U S A* **80**, 5369-73 (1983).

9. Evans, R.P., Jr. & Macrina, F.L. *Streptococcal* R plasmid pIP501: endonuclease site map, resistance determinant location, and construction of novel derivatives. *J Bacteriol* **154**, 1347-55 (1983).

10. Scheich, C., Niesen, F.H., Seckler, R. & Bussow, K. An automated in vitro protein folding screen applied to a human dynactin subunit. *Protein Sci* **13**, 370-80 (2004).

11. Arends, K. Ph.D thesis, Technical University Berlin (2010).

12. Lupas, A., Van Dyke, M. & Stock, J. Predicting coiled coils from protein sequences. *Science* **252**, 1162-4 (1991).
